# Trend in cancer incidence in Mato Grosso and its health regions, Brazil, 2001–2018

**DOI:** 10.1186/s13690-025-01503-9

**Published:** 2025-04-01

**Authors:** Marco Aurélio Bertúlio das Neves, Noemi Dreyer Galvão, Fernanda Cristina da Silva de Lima, Júlio Fernando Pinto Oliveria, Sancho Pedro Xavier, Kátia Moreira da Silva, Ádila de Queiroz Neves Almeida, Ageo Mário Cândido da Silva

**Affiliations:** 1 State Secretary of Health of Mato Grosso, Cuiabá, Mato Grosso Brazil; 2 Federal University of Mato Grosso - Institute of Collective Health, Av. Fernando Correa da Costa, nº 2367 - Bairro Boa Esperança, Cuiabá, MT 78060-900 Brazil; 3 Fundação do Câncer do Rio de Janeiro, Rio de Janeiro (RJ), Brasil; 4 Instituto Nacional de Câncer (INCA, Rio de Janeiro (RJ), Rio de Janeiro (RJ), Brasil

**Keywords:** Incidence, Trends, Neoplasms, Epidemiology

## Abstract

**Background:**

Given the lack of published evidence on cancer incidence trends in Mato Grosso disaggregated by health regions, this study aimed to analyze the temporal trends in cancer incidence across the health regions of Mato Grosso.

**Methods:**

Time series study that used data from the Population-Based Cancer Registry of Mato Grosso (2001–2018) to analyze cancer incidence trends. Age-standardized incidence rates were calculated and analyzed by year, sex, health regions, and primary cancer sites. Trends were estimated using the Joinpoint regression method, evaluating annual percentage changes (APC) and the average annual percentage change (AAPC) with a confidence interval of 95.0%.

**Results:**

Among men, an increasing trend was observed for prostate cancer in the state (APC: 2.6) from 2001 to 2013, as well as in the Baixada Cuiabana (AAPC: 3.5) and Middle North (APC: 5.5) regions from 2001 to 2015, having oscillated in three other regions and decreasing for lung cancer in the state (APC: -4.2) during 2001–2009 and 2012–2018, in Baixada Cuiabana (AAPC: -2.4), and Garças Araguaia (AAPC: -3.8), and cancers of the oral cavity (AAPC: -2.3) and stomach (AAPC: -3.5) in the state. Among women, a decreasing trend was observed for cervical cancer (AAPC: -6.8) both in the state and in all regions reporting cases. A decreasing trend was also noted for breast cancer in the state (APC: -3.6) from 2001 to 2009, with an increasing trend in the Southwest (AAPC: 5.8) and Araguaia Xingu (AAPC: 5.8) regions. Lung cancer showed a decreasing trend in the state (APC: -2.7) from 2001 to 2009, while thyroid cancer exhibited an increasing trend (AAPC: 6.7).

**Conclusion:**

By providing detailed information on cancer incidence trends by health region, this study underscores the need for region-specific interventions tailored to the unique magnitude of the cancer burden in each area.

**Supplementary Information:**

The online version contains supplementary material available at 10.1186/s13690-025-01503-9.

**Table:** 

Text box 1. Contributions to the literature	
In Brazil, cancer is the second leading cause of death, with an increasing trend expected in the coming years, surpassing the average in some Latin American countries. In the population of the state of Mato Grosso, located in the Brazilian Amazon, the impacts of cancer are still not well understood. In some municipalities in this region, cancer is the leading cause of death. Underreporting and limited access to specialized healthcare services are significant challenges. This study provides an important contribution to improving the prevention of the most prevalent cancers in the region, as well as enhancing early diagnosis and treatment, and reducing cancer-related morbidity and mortality.	

## Introduction

Cancer is a global health issue, ranking among the leading causes of death and posing a significant barrier to increasing life expectancy in countries worldwide [1]. In 2022, world cancer estimates reported approximately 19.9 million new cases and 9.7 million deaths, with both trends rising. In that same year, the most common cancers in men, excluding non-melanoma skin cancers, were lung cancer (32.1/100,000), prostate cancer (29.4/100,000), colorectal cancer (21.9/100,000), and stomach cancer (12.8/100,000). For women, breast cancer was the most prevalent (46.8 per 100,000), followed by lung cancer (16.2 per 100,000), colorectal cancer (15.2 per 100,000), and cervical cancer (14.1 per 100,000) [1].

In Brazil, estimates for each year of the three-year period (2023–2025) project 704,000 new cancer cases. Non-melanoma skin cancer is the most common (220,000 cases), followed by breast cancer (74,000), prostate cancer (72,000), colorectal cancer (46,000), lung cancer (32,000), and stomach cancer (21,000). In the Central-West region, where the state of Mato Grosso is located, 35,500 cases are estimated, with 8,000 in the state. The most common cancers in Mato Grosso, excluding non-melanoma skin cancer, are prostate, lung, colorectal, stomach, and oral cavity cancers in men, and breast, lung, colorectal, cervical, and thyroid cancers in women [2].

Trend analysis is an important tool for identifying cancer control priorities. However, its effective use requires a long historical series to detect changes over time [3–6]. Despite the research conducted so far, the incidence of these cancers disaggregated by health regions in Mato Grosso remains unknown. The state encompasses geographically diverse areas with significant regional inequalities and distinct risk factors contributing to the carcinogenesis process [7, 8]. Disaggregated analyses at smaller geographical units help identify different conditions within the same region, thereby supporting the planning of cancer prevention and control measures, as well as the implementation of policies to combat the disease [9].

In Brazil, one of the key tools for cancer surveillance is the population-based cancer registries (RCBP), which collect, analyze, and provide cancer incidence data for a population within specific coverage areas. These registries enable a better understanding of the disease’s occurrence within the territory they cover [10]. Based on information from cancer registries, it is possible to identify priority areas for cancer care in smaller geographical units [11, 12]. This study aimed to analyze the temporal trends of age-adjusted incidence rates for the main types of cancer by sex and Health Regions in Mato Grosso from 2001 to 2018.

## Methods

### Study design and data source

This is a time series study that utilized data from the Population-based Cancer Registries (RCBP) of the interior of Mato Grosso and Cuiabá, which were subsequently unified and renamed RCBP Mato Grosso, which examined the most incident types of cancer, excluding non-melanoma skin cancer, by sex, in the health regions of the state of Mato Grosso from 2001 to 2018. The incidence information is available at https://www.gov.br/inca/pt-br/assuntos/cancer/numeros/registros/base-populacional. Data sources include medical and laboratory institutions that diagnose cancer and institutions responsible for mortality records.

## Setting of the study

Mato Grosso, located in the Central-West Region of Brazil, spans an area of 903,208.36 km² and had a population of 3,658,649 in 2022. It is one of the nine Brazilian states belonging to the Legal Amazon, encompassing approximately two-thirds of the country’s territory. The state’s economy is dependent on agribusiness, with agricultural commodities forming the basis of its production [13]. Mato Grosso consists of 142 municipalities, which in the health sector are aggregated into 16 health regions: Alto Tapajós, Baixada Cuiabana, Araguaia Xingu, North Araguaia Karajá, North Center, Garças Araguaia, Middle Araguaia, Middle North, North, Northwest, West, Southwest, South, Teles Pires, Vale do Arinos, and Vale do Peixoto [14]. These regions vary in demographic, socioeconomic, and healthcare service availability [8, 15]. Specialized oncology care services are available in only three of these regions, with a higher concentration in the capital [16] .

## Selection criteria

The selection criteria included individuals residing in the coverage area of the RCBP Mato Grosso, with a confirmed diagnosis of malignant neoplasia through anatomopathological, cytological, hematological examinations, surgical exploration, imaging, clinical examination, autopsy, or other diagnostic means with medical opinion [17]. The most incident primary tumor sites by sex in the state of Mato Grosso were selected based on epidemiological relevance: oral cavity (C00-10), stomach (C16), colon and rectum (C18-C21), prostate (C61), lung (C33-C34), female breast (C50), cervix (C53), and thyroid gland (C73). All cases of benign neoplasms and those with uncertain or unknown behavior (D00-D48), were excluded. Tumor classification was based on the third edition of the International Classification of Diseases for Oncology (ICD-O3) [18], and was converted to the tenth revision of the International Statistical Classification of Diseases and Related Health Problems (ICD-10) [19, 20].

## Data quality

The percentages of microscopic verification (%MV), deaths reported solely by Death Certificates Only (%DCO), and the mortality/incidence ratio (M/I) were evaluated according to the criteria of the International Agency for Research on Cancer (IARC) [21]. In this study, the following quality parameters were considered: %MV above 70% and %DCO between 10% and 12% [21, 22].

### Data analysis

Crude and age-adjusted incidence rates per 100,000 inhabitants were calculated for each year from 2001 to 2018. The denominator used was composed of census population estimates (2010) and intercensal estimates (2001 to 2018) by age groups, obtained from the Department of Informatics of the Unified Health System (DATASUS) and provided by the Brazilian Institute of Geography and Statistics (IBGE) [23].

Specific crude rates were calculated for each age group, using 5-year intervals for children and adolescents (0 to 19 years) and 10-year intervals for adults (20 years or older). The adjusted incidence rates were standardized using the direct method, with the world standard population proposed by Segi and modified by Doll. The five most frequently adjusted rates were selected for analysis. The data were compiled and analyzed using Microsoft Excel version 16.77.1.

Trend analyses of incidence rates by health regions were limited to the two most common cancers in men and women. The Joinpoint regression model was used, which adjusts linear trends and changes in these trends (inflection points) on a logarithmic scale, with the calendar year as the regressor variable. Estimates of values were obtained using the statistical goodness-of-fit test, applying the Monte Carlo permutation method. The direction and magnitude of the trends were assessed using the Annual Percentage Change (APC) and the Average Annual Percentage Change (AAPC) [24, 25]. The trends presented in this study were statistically significant at the 95% confidence level (95% CI). Statistical analyses were performed using the Joinpoint Regression Program, version 4.9.1.0.

## Results

Between 2001 and 2018, there were 63,392 new cases of cancer, excluding non-melanoma skin cancer, with 33,878 cases in men (53.4%) and 29,514 in women (46.6%). The most common cancers in men were prostate (29.2%), lung (8.8%), stomach (7.1%), colon and rectum (6.8%), and oral cavity (5.0%). Among women, the most common cancers were breast (26.3%), cervix (13.3%), colon and rectum (7.7%), lung (5.6%), and thyroid (4.2%) (Table 1).

**Table 1:** Proportional distribution of the five most frequent types of cancer by sex in the state of Mato Grosso, Brazil, 2001–2018

Primary tumor location	Gender					Total
	Male		Female			
	Freq Absolute	Freq Relative	Freq Absolute	Freq Relative		
Prostate	9.904	29,2	-		-	9.904
Lungs	2.994	8,8	1.642		5,6	4.636
Colon and rectum	2.296	6,8	2.285		7,7	4.581
Breast	-	-	7.754		26,3	7.754
Cervix	-	-	3.915		13,3	3.915
Stomach	2.410	7,1	1.191		4,0	3.601
Oral cavity	1.696	5,0	505		1,7	2.201
Thyroid	298	0,9	1.232		4,2	1.530
Other Locations	14.280	42,2	10.990		37,2	25.270
Non-melanoma skin	11.032	-	10.530		-	21.562
All neoplasms	44.910		40.044			84.954
All neoplasms excluding non-melanoma skin	33.878		29.514			63.392

Quality indicators by primary tumor location by sex are shown in Supplementary Information (SI-1). Overall, the quality of data for the main primary tumor locations was higher for females throughout the study period. Among males, a %MV above 70.0% was observed only for prostate, colorectal, and oral cavity cancers, while for females, it was achieved for all cancers except lung cancer. A %DCO rate below 12.0% was found only in females, specifically for breast, cervical, and thyroid cancers (SI-1).

Age-specific incidence rates for the five most frequent primary locations in men and women are presented in Supplementary Information (SI-2). Among men, incidence rates began to increase at age 40, with a steeper rise from age 60 onwards. Prostate cancer was particularly prominent, especially in older age groups, with an incidence rate of 55.04 per 100,000 in the 50–59 age group, escalating to 690.46 per 100,000 in men aged 80 or older. In females, breast and cervical cancers occurred at younger ages (from 30 years) and increased with age, while other cancers became more prevalent from age 50 (SI-2).

**Fig. 1:**
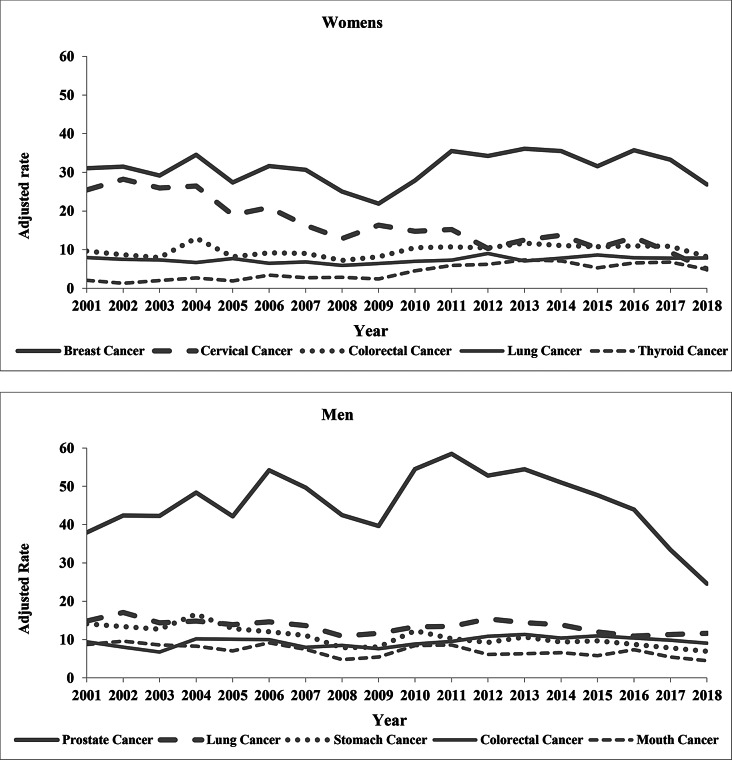
Age-standardized incidence rates (world standard population) per 100,000 men and women of the 5 most frequent primary locations in Mato Grosso, Brazil, 2001–2018

Prostate and lung cancers were the most common among men, followed by stomach, colorectal, and oral cavity cancers. In women, breast and cervical cancers were the most frequent, followed by colorectal, lung, and thyroid cancers. The age-standardized incidence rates for the state of Mato Grosso mirrored the pattern observed nationwide in Brazil (Fig. 1).

Figure 1. Age-standardized incidence rates (world standard population), per 100,000 men and women, of the 5 most frequent primary locations in Mato Grosso, Brazil, 2001–2018.

**Fig. 2:**
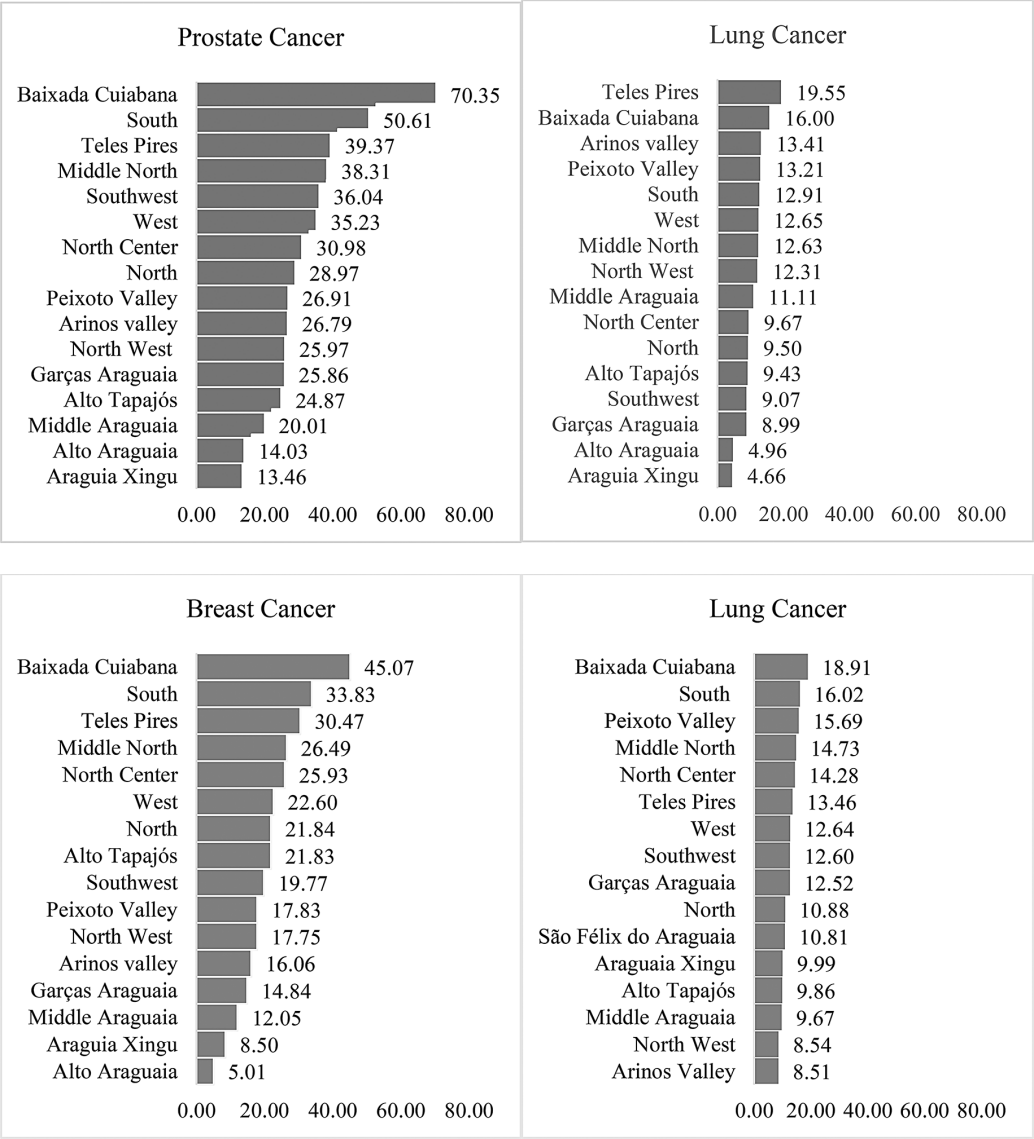
Age-standardized incidence rate of the two most frequent primary localizations by sex (World Standard Population, according to Health Region) in the state of Mato Grosso, Brazil, 2001–2018

The health regions of Baixada Cuiabana, South, Teles Pires, and Middle North had the highest rates for prostate and breast cancers. Cervical cancer showed the highest incidence rates in the regions of Baixada Cuiabana, South, Vale do Peixoto, Middle North, North Center, and Teles Pires. In men, lung cancer had the highest rates in Teles Pires, Baixada Cuiabana, Juara, Vale do Peixoto, South, West, and North Center (Fig. 2). In view of the great variability of the incidence rates observed between the regions, the Supplementary Information (SI-3) presents a table with the proportional distribution of the most frequent types of cancer by sex, health region and population of the study period.

Figure 2. Age-standardized incidence rate of the two most frequent primary localizations by sex (World Standard Population, according to Health Region) in the state of Mato Grosso, Brazil, 2001–2018.

In the analysis of APC in cancer incidence rates by sex, a rising trend was observed in the age-adjusted incidence rates for thyroid cancer in females, with an AAPC of 6.7%, and a significant decline for cervical cancer, with an AAPC of -6.8%. Other cancers analyzed exhibited statistically significant trends in specific periods. Female breast and lung cancers showed reductions, with APCs of -3.6% and − 2.7%, respectively, from 2001 to 2009.

Among males, reductions were observed in the incidence rates for stomach and oral cavity cancer, with AAPCs of -3.5% and − 2.3% respectively. In contrast, prostate cancer showed an increase of 2.6% AAPC from 2001 to 2013. Lung cancer exhibited a decline in two distinct periods: from 2001 to 2009, with an APC of -4.2%, and from 2012 to 2018, with an APC of -5.9%. Other cancers analyzed displayed statistically significant trends over shorter periods (Table 2).

**Table 2:** Cancer incidence trends, age-standardized, by world standard population, among men and women in the state of Mato Grosso, Brazil, 2001–2018

Primary tumor location	Trends 1		Trends 2		Trends 3		Trends 4		AAPC (95%CI)
	Period	APC (95%CI)	Period	APC (95%CI)	Period	APC (95%CI)	Period	APC (95%CI)	
	Men								
Prostate	2001–2013	2,6*(0,4;4,9)	2013–2018	-10,5(-21,3;1,8)	-	-	-	-	-1,4(-5,0;2,3)
Lungs	2001–2009	-4,2*(-6,3; -2,1)	2009–2012	8,7(-10,8;32,4)	2012–2018	-5,9*(-9,8; -1,9)			-2,7(-6,0;0,7)
Stomach	2001–2018	-3,5*(-5,0; -2,0)	-	-	-	-	-	-	-3,5*(-5,0; -2,0)
Colon and rectum	2001–2005	5,4(-6,5;18,9)	2005–2009	-6,1(-18;7,6)	2009–2013	10,2(-2,2;24,3)	2013–2018	-4,1(-11,3;3,6)	0,8(-3,7;5,6)
Oral cavity	2001–2018	-2,3*(-4,2; -0,4)	-	-	-	-	-	-	-2,3*(-4,2; -0,4)
	**Women**								
Breast	2001–2009	-3,6*(-5,7; -1,5)	2009–2012	12,7(-8,4;38,8)	2012–2018	-2,6(-5,9;0,8)	-	-	-0,6(-4,0;3,0)
Cervix	2001–2018	-6,8*(-8,0; -5,5)	-	-	-	-	-	-	-6,8*(-8,0;-5,5)
Colon and rectum	2001–2004	9,5(-6,3;28)	2004–2008	-8,8(-17,9;1,3)	2008–2011	13,9(-7,4;40,1)	2011–2018	-1(-3,3;1,4)	1,3(-3,0;5,9)
Lungs	2001–2009	-2,7*(-4,4; -1,0)	2009–2012	9,6(-8;30,7)	2012–2018	-0,9(-3,7;1,9)	-	-	-0,01(-2,9;3,0)
Thyroid	2001–2009	6,3(-0,3;13,4)	2009–2012	30,3(-2,1;73,6)	2012–2018	-3(-6,6;0,8)	-	-	6,7*(1,2;12,5)

*APC or AAPC values were considered statistically significant if *p* < 0.05. For rates equal to zero, calculation of the trend was not possible, and such instances are indicated with a dash (‘-‘) in the table

When analyzing each type of cancer by sex and health regions, at least one health region showed a significant trend in incidence for the level of significant at the 95% CI. A decline in the incidence trend for prostate cancer was observed, with an AAPC of -3.5%, specifically in the Baixada Cuiabana health region. Some regions showed statistically significant trends over short periods: increasing trends were observed in Garças Araguaia (2001–2003; 2003–2016), Southwest (2001–2015), South (2001–2016), and Middle North (2001–2015); decreasing trends were noted in Garças Araguaia (2016–2018), Southwest (2015–2018), and South (2012–2018). In men, a reduction in lung cancer incidence was also observed in the Baixada Cuiabana and Garças Araguaia health regions, with AAPCs of -2.4% and − 3.8% respectively. (Table 3).

**Table 3:** Prostate and lung cancer incidence trends, age-standardized, by world standard population, among men, by Health Region in the state of Mato Grosso, Brazil, 2001–2018

Health Region	Prostate Cancer								AAPC (95%CI)	Lung Cancer	
	Trends 1		Trends 2		Trends 3		Trends 4			Trends 1	
	Period	APC (95%CI)	Period	APC (95%CI)	Period	APC (95%CI)	Period	APC (95%CI)		Period	AAPC (95%CI)
Alto Tapajós	2001–2016	4,2(-3,1;12,1)	2016–2018	-72,4(-95,1;55,6)	-	-	-	-	-10,9(-26,6;8,2)	-	-
Baixada Cuiabana	2001–2012	0,8(-1,9;3,5)	2012–2018	-11,0*(-16,8; -4,9)	-	-	-	-	-3,5*(-6,1; -1,0)	2001–2018	-2,4*(-3,8; -0,9)
Garças Araguaia	2001–2003	98,5*(20,1;228,3)	2003–2016	3,4*(0,4;6,5)	2016–2018	-49,5*(-69,4; -16,4)	-	-	2,6(-4,9;10,8)	2001–2018	-3,8*(-6,5; -1,0)
West	2001–2014	4,9(-0,2;10,1)	2014–2018	-25,5(-44,6;0,2)	-	-	-	-	-3,2(-9,9;4,0)	2001–2018	-2,1(-5,5;1,5)
North	2001–2014	5,2(-0,8;11,6)	2014–2018	-21,8(-45,2;11,6)	-	-	-	-	-1,9(-10,0;7,0)	2001–2018	1,0(-4,3;6,7)
North Center	2001–2018	0,4(-3,4;4,3)	-	-	-	-	-	-	0,4(-3,4;4,3)	2001–2018	-3,1(-7,6;1,7)
Vale do Arinos	2001–2018	-1(-5,6;3,9)	-	-	-	-	-	-	-1,0(-5,6;3,9)	-	-
Northwest	2001–2018	2,8(-1,8;7,6)	-	-	-	-	-	-	2,8(-1,8;7,6)	2001–2018	-3,6(-7,8;0,7)
Vale do Peixoto	2001–2018	-0,8(-5,2;3,9)	-	-	-	-	-	-	-0,8(-5,2;3,9)	2001–2018	1,6(-2,5;5,9)
Southwest	2001–2015	9,9*(4,6;15,4)	2015–2018	-45,6*(-67,8; -8)	-	-	-	-	-2,9(-11,4;6,4)	-	-
South	2001–2006	15,2*(5,8;25,4)	2006–2009	-12,6(-40,1;27,7)	2009–2012	29,9(-11,1;89,7)	2012–2018	-9,5*(-15,1; -3,5)	2,9(-5,3;11,9)	2001–2018	-0,9(-3,1;1,4)
Teles Pires	2001–2005	16,7(-7,9;47,8)	2005–2008	-24,7(-64,3;58,9)	2008–2015	5,6(-6,9;19,8)	2015–2018	-23,3(-47,2;11,4)	-3,7(-16,1;10,4)	2001–2018	-0,2(-3,2;3,0)
Middle North	2001–2015	5,5*(1,2;10,1)	2015–2018	-32,7(-57;5,5)	-	-	-	-	-2,5(-9,9;5,4)	2001–2018	-2,4(-4,7;0,0)

*APC or AAPC values were considered statistically significant if *p* < 0.05. For rates equal to zero, calculation of the trend was not possible, and such instances are indicated with a dash (‘-‘) in the table

For female breast cancer, an increase in adjusted rates was observed in both the Southwest and Araguaia Xingu regions, with an AAPC of 5.8%. Cervical cancer trends showed a reduction across nine health regions: Baixada Cuiabana, Garças Araguaia, West, North Center, Northwest, Vale do Peixoto, South, Teles Pires, and Middle North. (Table 4). There were situations in which the trends were not calculated, in view of the existence of rates with zero values in some year of the period. These situations are identified in the Tables 3 and 4 by the absence of (-) values. The Supplementary Information (SI-4) presents figures that demonstrate the variability of incidence rates by type of cancer, sex, region and year.

**Table 4:** Trends in breast and cervical cancer incidence, age-standardized, by world standard population, among women, by Health Region in the state of Mato Grosso, Brazil, 2001–2018

	Female breast cancer			Cervical cancer				
Health Region	Trends 1		AAPC (95%CI)	Trends 1		Trends 2		AAPC (95%CI)
	Period	APC (95%CI)		Period	APC (95%CI)	Period	APC (95%CI)	
Alto Tapajós	2001–2018	-0,01(-4,4;4,6)	-0,01(-4,4;4,6)	-	-			-
Baixada Cuiabana	2001–2018	0,9(-0,6;2,3)	0,9(-0,6;2,3)	2001–2018	-7,1*(-9,1; -5,2)			-7,1*(-9,1; -5,2)
Garças Araguaia	2001–2018	-0,1(-6,3;6,6)	-0,1(-6,3;6,6)	2001–2018	-9,6*(-14,6; -4,3)			-9,6*(-14,6; -4,3)
West	2001–2018	-1,5(-3,5;0,6)	-1,5(-3,5;0,6)	2001–2018	-8,3*(-11,7; -4,7)			-8,3*(-11,7; -4,7)
North	2001–2018	-0,1(-4,3;4,3)	-0,1(-4,3;4,3)	-	-			-
North Center	2001–2018	-0,8(-4,1;2,6)	-0,8(-4,1;2,6)	2001–2018	-4,4*(-7,7; -1,0)			-4,4*(-7,7; -1,0)
Northwest	2001–2018	-1,7(-5,3;2)	-1,7(-5,3;2)	2001–2018	-10,0*(-14,5; -5,4)			-10,0*(-14,5; -5,4)
Vale do Peixoto	2001–2018	0,8(-4,0;5,8)	0,8(-4,0;5,8)	2001–2018	-7,6*(-12,6; -2,3)			-7,6*(-12,6; -2,3)
Southwest	2001–2018	5,8*(0,1;11,9)	5,8*(0,1;11,9)	-	-			-
Araguaia Xingu	2001–2018	5,8*(1,6;10,1)	5,8*(1,6;10,1)	-	-			-
South	2001–2018	2,3(-0,5;5,2)	2,3(-0,5;5,2)	2001–2018	-6,2*(-8,4; -4,0)			-6,2*(-8,4; -4,0)
Teles Pires	2001–2018	-1,3(-3,9;1,4)	-1,3(-3,9;1,4)	2001–2018	-8,4*(-12,1; -4,6)			-8,4*(-12,1; -4,6)
Middle North	2001–2018	1(-2,7;4,9)	1(-2,7;4,9)	2001–2016	-4,3*(-8,0; -0,5)	2016–2018	49,8(-80,3;27,7)	-11,3*(-20,1; -1,5)

*APC or AAPC values were considered statistically significant if *p* < 0.05. For rates equal to zero, calculation of the trend was not possible, and such instances are indicated with a dash (‘-‘) in the table

## Discussion

This study presented trends in age-standardized incidence rates of the five most common types of cancer for both sexes in the state of Mato Grosso, as well as the two most common types in men and women, according to health regions, from 2001 to 2018. During this period, the most common cancers in the state, excluding non-melanoma skin cancer, were prostate, breast, lung, colorectal, and cervical cancers. This pattern differs from global and Latin American and Caribbean profiles, where breast and stomach cancers rank as the most and fifth most common, respectively [1]. In the Central-West region, colorectal cancer is estimated to have a higher incidence than lung cancer [2]. However, the cancer profile in Mato Grosso aligns with studies conducted in Goiânia, Goiás, and São Paulo [26, 27]. The highest incidence rates were observed in the North Center, South, Teles Pires, and Middle North health regions.

In men, prostate and lung cancers were the most common, while in women, breast and cervical cancers were the most frequent. The prevalence of prostate and breast cancers is consistent with global and Brazilian patterns. The cancer occurrence profile in Mato Grosso possibly reflects the concept of epidemiological transition, which explains changes in disease patterns due to factors such as infections, socioeconomic development, and unhealthy lifestyle habits [1, 9]. Regarding changes in demographic, socioeconomic and infectious profiles in Brazil and Mato Grosso, several studies point to an epidemiological transition that reflects the process of change in the predominant diseases over time. In particular, Brazil has experienced a significant reduction in mortality from infectious and parasitic diseases, which were dominant in past decades, while chronic non-communicable diseases, such as cancer and cardiovascular diseases, have become more prevalent [28, 29].

In the regional context, Mato Grosso state reflects these changes, but with a transition that occurs unevenly between regions. The study of the epidemiological profile in Brazil showed that while the southern and southeastern regions experience a higher prevalence of chronic diseases, the North and Midwest regions, including Mato Grosso, still face challenges related to infectious and parasitic diseases [30]. Given that agribusiness is the main economic driver in the state, this industry likely influences the cancer occurrence profile [31].

The great regional variability in the occurrence of cancer cases over the period studied deserves to be highlighted. This situation is possibly due to the fact that, in each health region, the health system works in different ways, and the diagnostic network is concentrated in municipalities with higher density, thus influencing the quality of the data recorded [32].

The variability observed in the Garças Araguaia region for prostate cancer is a good example. This situation is due to the large difference in the number of cases observed in the first two and last two years of the study period in relation to the other years. Possibly, three factors justify this situation, namely: the RCBP was implemented in Mato Grosso in 1999 and the registration of cases did not yet occur properly in the initial years; the closure in 2016 of a state reference Health Unit for cancer care; and the state service responsible for Cancer Surveillance did not carry out an active search for cases in the last two years of the study period. However, the possibility of underreporting cannot be disregarded.

The quality indicators used to assess the RCBP data are closely related to the number of cases and the availability of health services for cancer diagnosis and treatment within the registry’s coverage area [33]. The IARC evaluates the quality of RCBP databases, but the CI5 XII (IARC) publication currently classifies these databases based on information quality without specifying exact values for quality indicators [33, 34]. In low- and middle-income countries (LMICs), these indicators pose challenges due to incomplete data, the need for active case finding, and the sustainability of registries to monitor cancer rates and trends over the long term. Despite not meeting all quality control standards, the dissemination and analysis of cancer registry information remain valuable. They can provide insights into healthcare system deficiencies and support planning for cancer control actions [9, 22, 33].

Regarding the quality of RCBP data, this study found that data quality indicators for primary tumor sites were generally better for females, with %MV above 70.0% observed in four of the five most common cancers in women and %DCO below 12.0% in three. However, the %DCO for lung and stomach cancers was notably higher compared to a study conducted in Mendoza, Argentina [35] Tracking cases reported through death certificates could be a way to improve data quality by reducing %DCO.

An important differential of the State in relation to other Brazilian states is in the territorial extension and vigor of the economy based on agricultural production. The health regions are very distinct demographically, socially, economically and in the structure and access to the health service network for cancer care. In health regions that have a predominance of chemical agricultural production dependent on agribusiness, there is evidence of high exposure to carcinogenic agents and ineffective performance of cancer surveillance services.

In analyzing cancer incidence by health region, the highest age-adjusted rates were concentrated in the four most developed, populous, and socioeconomically advanced health regions, which represent 57.0% of the state’s population. Mato Grosso showed an increasing trend for prostate cancer between 2001 and 2013 (APC: 2.6%), with significant increases in specific regions such as Garças Araguaia (2001–2003 and 2003–2016), Southwest, Middle North (2001–2015), and South (2001–2006). Conversely, decreasing trends were observed in the Baixada Cuiabana, Garças Araguaia (2016–2018), Southwest (2015–2018), and South (2012–2018) regions. A similar reduction was noted in Cuiabá and Várzea Grande (2006–2016) [37]. In Brazil, an increasing trend was observed from 2001 to 2016 (AAPC: 0.47). A global study on prostate cancer incidence from 2000 to 2019 found increases in 73.0% of countries, stability in 28.1%, and decreases in 10.1%, with the highest rates in countries with a high or very high Human Development Index (HDI) [7, 38]. The rise in prostate cancer incidence may be attributed to advances in diagnostic methods, improvements in data quality, increased life expectancy [39], modifiable risk factors related to environmental and lifestyle factors such as diet and physical activity, non-modifiable factors such as age, hormonal aspects, and genetics [1], challenges in accessing healthcare, local screening and diagnostic practices, and differences in therapeutic approaches and care quality [40–43]. Men in low socioeconomic settings may face higher risks compared to those in other settings [41]. Conversely, the decrease or stabilization in incidence may reflect a reduction in screening due to a lack of promotion for the prostate-specific antigen (PSA) test by some health agencies [44]. The non-recommendation by the Brazilian Ministry of Health is due to the lack of scientific evidence so far that this practice brings more benefits than risks, as well as, there is no evidence that PSA screening reduces overall mortality for men of any age and consistent evidence that screening and active treatment lead to harm [45]. Despite the utilization of digital rectal examination (DRE) in clinical practice to detect prostate cancer, there is currently no evidence indicating that DRE alone or in conjunction with the PSA test results in a reduction in prostate cancer mortality [46]. To increase this proportion, it is essential to improve awareness of men’s health and the importance of screening, as well as to ensure that health services are accessible and appropriate to the needs of this population.

Although lung cancer is the most common cancer in men worldwide, its incidence has been declining globally and in Brazil since the 1980s. It is estimated to be the third most common cancer in men in the Central-West region in 2023 and to account for 30.0% of all cancers in men in Mato Grosso [2]. This study found a decreasing trend in lung cancer incidence in men from 2001 to 2009 (APC: -4.2) and 2012–2018 (APC: -5.9), with reductions also observed in the Baixada Cuiabana (AAPC: -2.4) and Garças Araguaia (AAPC: -3.8) regions. Similar findings were observed in studies conducted in the Greater Cuiabá area (AAPC: -2.2) [37, 47]. Despite this trend, eight health regions, representing 78.2% of the state’s population, had incidence rates during the study period that exceeded the estimated rate for the state in 2023 (11.98/100,000 men) [2]. Key factors contributing to this trend include reductions in smoking and exposure to secondhand smoke, as approximately 85.0% of diagnosed cases are associated with tobacco use [48]. Brazil’s tobacco control policies, focusing on legislative and social changes, have effectively contributed to smoking cessation rates [49]. According to the National Health Survey (PNS), in Brazil, there was a reduction in the prevalence of adult male smokers from 18.9% in 2013 to 15.9% in 2019 [50], and in 2023, this percentage was 10.2% [51], while the WHO Global Report on Trends in the Prevalence of Tobacco Use 2000–2025, points out that in 2020, 36.7% of the adult population in the world used tobacco.

Additionally, occupational and environmental exposure to carcinogenic agents, particularly in an agriculturally chemical-dependent economy, plays a significant role.A prevalence study of occupational and environmental exposures among patients in Mato Grosso found pesticides to be the most common exposure, affecting around 20.0% of patients [36]. Despite the decreasing trend found, the absence of planned and systemic interventions by state public management since the early 2010s remains a concern.

Even though, female breast cancer has surpassed lung cancer as the most diagnosed cancer worldwide, excluding non-melanoma skin cancer, the trend in Mato Grosso showed a decrease from 2001 to 2009 (APC: -3.6) with an increase in the Southwest and Araguaia Xingu regions (AAPC: 5.8). This state-level trend aligns with observations from developed countries since the 2000s [1]. However, a shorter historical analysis found an increasing trend from 2009 to 2016 in the state and a decreasing trend in Baixada Cuiabana from 2000 to 2009 [7]. Since the 2000s, increasing breast cancer incidence has been noted in developing countries, including South America, Asia, and Africa [1]. The rise in incidence is linked to aging, behavioral, environmental, and reproductive risk factors, as well as economic development, improved healthcare services, and enhanced early detection through widespread mammographic screening, recommended in Brazil for women aged 50 to 69 years [1, 52]. Notable behavioral risks include alcohol consumption, overweight, physical inactivity, and exposure to ionizing radiation [53–55]. Among reproductive factors, fewer children, shorter periods of exclusive breastfeeding, and delayed childbirth are significant [1]. Regarding the availability of services and improvements in early detection, a study conducted in São Paulo and Campinas (Brazil) found a statistically significant association between suspected breast cancer identified by Primary Health Care (PHC) and economic and cancer care variables, such as breast examination at PHC before referral to Specialized Care (SC), initial mammogram requests by PHC, and continuity of care in PHC post-treatment [56].

A global trend study found increasing rates of cancers associated with socioeconomic development, such as breast and lung cancer, particularly in low- and middle-income countries [57]. Studies conducted in the Barretos region and São Paulo districts support this association, as breast cancer incidence rates in São Paulo were 30% higher than in Barretos, particularly in districts with high socioeconomic levels [12]. The varying trends in breast cancer incidence across regions may reflect differences in exposure to risk factors, the availability and access to healthcare services, and the differing use of early detection procedures [56, 58, 59]. Although this study did not identify statistical significance for the trend in breast cancer incidence in the state from 2009 to 2018, the ratio of mammograms in women aged 50 to 69 years has always been lower than that of the Central-West region and the country. This ratio in the state in 2021 was the same as in 2008 (0.08) [60]. These results demonstrate great weakness in the actions for early detection of this cancer, calling into question whether the downward trend detected in the period from 2001 to 2009 is due to the secondary prevention actions implemented.

Globally, cervical cancer is the fourth most common cancer among women, and the third most common in Brazil and the Central-West region [2, 61]. In Mato Grosso, it is the second most common, excluding non-melanoma skin cancer. The study identified a decreasing trend in cervical cancer across the state and in the nine health regions with calculable rates: Baixada Cuiabana, Garças Araguaia, West, North Central, Northwest, Vale do Peixoto, South, Teles Pires, and Middle North. A decreasing trend has been observed in recent decades in studies conducted globally and in Brazil. Notable reductions have been found in Latin America, Asia, and Brazilian cities such as Belém, Palmas, Salvador, Fortaleza, Distrito Federal, Belo Horizonte, Grande Vitória, Curitiba, São Paulo, Porto Alegre, Campinas, and Goiânia [62, 63]. A different situation, particularly among young women, has been observed in some countries in Eastern Europe, Asia, and Central Europe. The highest incidences have been reported in Sub-Saharan African and Southeast Asian countries [64, 65]. Although the main risk factor for cervical cancer is persistent infection with an oncogenic type of Human Papillomavirus (HPV), factors related to immunity and genetics can influence the mechanisms that determine the persistence or regression of the infection, as well as associated cofactors such as age, smoking, education, and multiparity [48, 66]. The incidence of this cancer can be reduced through vaccination against oncogenic HPV types, screening through cytopathological examination, and treatment of precursor lesions in women aged 25 to 64 years [48]. The Supplementary Information **(SI-5)** presents in a systematized way the primary and secondary prevention measures by type of cancer.

Despite recent reductions in cervical cancer incidence in Brazil and Mato Grosso, HPV vaccination coverage has declined, and the ratio of cytopathological exams to the female population aged 25 to 64 years has decreased. In 2019, 87.08% of Brazilian girls aged 9 to 14 received the first vaccine dose, dropping to 75.81% in 2022. Coverage among boys fell from 61.55% in 2019 to 52.16% in 2022 [67]. The ratio of cytopathological exams to one-third of the female population decreased from 0.56 in 2008 to 0.29 in 2021 [60].

The limitations of the present study include the possibility of underreporting, filling and coding errors, and the incompleteness of certain variables, which may have impacted the analysis and interpretation of the results. If cancer cases over the observed period were constant, it would be possible to obtain a more detailed view of the variations in incidence rates. However, this was not observed, because there is regional variability and, in some periods, there were no cases of cancer to be analyzed, so it was not possible to apply the method. Although this study identified temporal associations, it was not possible to identify causality because underlying factors can influence both exposure and outcome, and in the trends identified it was not possible to assess the influence of seasonal or socioeconomic changes. Nevertheless, the study stands out as one of the first to analyze trends in the incidence rates of the most frequent cancers in both sexes in Mato Grosso, identifying significant regional variations. Additionally, it highlights the local epidemiological profile, reflecting the epidemiological transition and the relationship between socioeconomic development and the prevalence of cancers associated with infections and unhealthy lifestyle habits. Furthermore, the analysis of data quality revealed better completeness for female cancers, which is useful for identifying healthcare system deficiencies and guiding control action planning, particularly in regions with higher rates.

## Conclusion

This study provided information on the temporal trends of the most incident cancers in Mato Grosso, using the largest historical series of any state in the Legal Amazon. By producing trend behavior information disaggregated by health regions, it demonstrated the relevance of the RCBP for understanding the magnitude, defining priorities, and developing strategies for cancer promotion, prevention, and control. The results underscore the necessity for implementing targeted interventions and enhancing the availability and quality of healthcare services in Mato Grosso. This should be tailored to address the specific trends and severity of cancer in each health region within the state.

## Electronic supplementary material

Below is the link to the electronic supplementary material.

Supplementary Material 1

Supplementary Material 2

Supplementary Material 3

Supplementary Material 4

Supplementary Material 5

## Data Availability

The datasets analyzed during the current study are available in the repositories: from the population-based registries of INCA at https://www.gov.br/inca/pt-br/assuntos/cancer/numeros/registros/base-populacional and from the Department of Informatics of the Unified Health System – DATASUS at http://tabnet.datasus.gov.br/cgi/deftohtm.exe?ibge/cnv/popmt.def.
