# Supplementary material for: Trend in cancer incidence in Mato Grosso and its health regions, Brazil, 2001–2018
Source: Arch Public Health. 2025 Apr 1;83:87. doi: 10.1186/s13690-025-01503-9 (PMC11960033; doi:10.1186/s13690-025-01503-9)
Supplement: Supplementary file 1 — Supplementary Material 1 [file 13690_2025_1503_MOESM1_ESM.docx]

**SI-1.** Quality indicators, by primary tumor location, by gender, Mato Grosso, Brazil, 2001 to 2018

| **Primary tumor location** | **Nr.** | **VM (%)** | **DCO (%)** | **M/I (%)** |
| --- | --- | --- | --- | --- |
|  | **Men** |  |  |  |
| Prostate | 9.904 | 82,4 | 15,6 | 30,8 |
| Lungs | 2.994 | 44,4 | 51,3 | 95,2 |
| Stomach | 2.410 | 65,9 | 31,3 | 74,4 |
| Colon and rectal | 2.296 | 79,1 | 16,9 | 47,1 |
| Oral cavity | 1.696 | 84,9 | 12,2 | 46,4 |
|  | **Women** |  |  |  |
| Breast | 7.754 | 90,4 | 6,9 | 28,4 |
| Cervix | 3.915 | 88,5 | 8,2 | 35,4 |
| Colon and rectal | 2.285 | 81,1 | 16,5 | 45,9 |
| Lungs | 1.642 | 46,8 | 49,0 | 91,0 |
| Thyroid | 1.232 | 94,5 | 2,9 | 5,5 |

**Note:** Microscopic Verification (MV); Death Certificate Only (DCO); Mortality and Incidence Ratio (M/I)
