# Supplementary material for: Trend in cancer incidence in Mato Grosso and its health regions, Brazil, 2001–2018
Source: Arch Public Health. 2025 Apr 1;83:87. doi: 10.1186/s13690-025-01503-9 (PMC11960033; doi:10.1186/s13690-025-01503-9)
Supplement: Supplementary file 2 — Supplementary Material 2 [file 13690_2025_1503_MOESM2_ESM.docx]

**SI-2.** Specific incidence rates by age group for the 5 most frequent primary localizations, per 100,000 women and men, Mato Grosso, Brazil, 2001 to 2018

| **Primary tumor location** | **Age group (years)** | | | | | | | | | | |
| --- | --- | --- | --- | --- | --- | --- | --- | --- | --- | --- | --- |
|  | **0-04** | **05-09** | **10-14** | **15-19** | **20-29** | **30-39** | **40-49** | **50-59** | **60-69** | **70-79** | **80 ou +** |
| **Women** | | | | | | | | | | | |
| Breast | 0,00 | 0,00 | 0,04 | 0,12 | 2,98 | 21,64 | 61,75 | 91,46 | 106,80 | 123,18 | 141,50 |
| Cervix | 0,00 | 0,00 | 0,04 | 0,44 | 4,88 | 16,98 | 28,81 | 38,59 | 44,35 | 57,88 | 48,29 |
| Colon and rectal | 0,00 | 0,00 | 0,00 | 0,12 | 0,97 | 3,41 | 9,64 | 25,90 | 39,96 | 71,23 | 91,95 |
| Lungs | 0,04 | 0,00 | 0,08 | 0,08 | 0,34 | 0,99 | 4,97 | 16,36 | 34,71 | 67,61 | 76,42 |
| Thyroid | 0,00 | 0,04 | 0,33 | 0,80 | 3,51 | 6,33 | 9,61 | 9,68 | 9,95 | 9,56 | 5,88 |
| **Men** | | | | | | | | | | | |
| Prostate | 0,00 | 0,00 | 0,00 | 0,00 | 0,10 | 0,66 | 5,12 | 55,04 | 250,09 | 501,76 | 690,46 |
| Lungs | 0,00 | 0,00 | 0,00 | 0,04 | 0,27 | 0,88 | 6,34 | 26,10 | 69,32 | 125,21 | 136,23 |
| Stomach | 0,08 | 0,12 | 0,00 | 0,08 | 0,35 | 1,74 | 7,48 | 20,88 | 47,67 | 92,83 | 113,15 |
| Colon and rectal | 0,04 | 0,00 | 0,04 | 0,19 | 1,09 | 3,10 | 9,63 | 20,22 | 43,44 | 72,12 | 79,87 |
| Oral cavity | 0,12 | 0,00 | 0,00 | 0,12 | 0,41 | 1,28 | 8,87 | 22,52 | 29,59 | 37,90 | 36,39 |
