# Supplementary material for: Trend in cancer incidence in Mato Grosso and its health regions, Brazil, 2001–2018
Source: Arch Public Health. 2025 Apr 1;83:87. doi: 10.1186/s13690-025-01503-9 (PMC11960033; doi:10.1186/s13690-025-01503-9)
Supplement: Supplementary file 3 — Supplementary Material 3 [file 13690_2025_1503_MOESM3_ESM.docx]

**SI-3.** Proportional distribution of the most frequent types of cancer by Health Region, Mato Grosso, Brazil, 2001 - 2018

| **Health Region** | **Breast** | **Cervix** | **Female Population** | **Prostate** | **Lung** | **Male Population** |
| --- | --- | --- | --- | --- | --- | --- |
|  | **Cases** | |  | **Cases** | |  |
| Middle Araguaia | 70 | 65 | 696.650 | 117 | 65 | 769.155 |
| Alto Tapajós | 174 | 83 | 860.142 | 204 | 81 | 949.067 |
| Baixada Cuiabana | 3.691 | 1.602 | 8.369.018 | 4.484 | 1.055 | 8.202.094 |
| Garças Araguaia | 158 | 139 | 1.067.156 | 279 | 96 | 1.098.969 |
| West | 371 | 214 | 1.653.021 | 579 | 204 | 1.721.137 |
| North | 130 | 67 | 612.563 | 180 | 58 | 658.570 |
| North Center | 194 | 110 | 808.158 | 260 | 84 | 903.437 |
| Arinos Valley | 62 | 32 | 455.688 | 106 | 56 | 497.522 |
| Northwest | 163 | 86 | 1.134.531 | 218 | 112 | 1.263.035 |
| Vale do Peixoto | 138 | 123 | 840.030 | 198 | 101 | 902.147 |
| Southwest | 163 | 112 | 965.200 | 291 | 76 | 1.019.515 |
| Araguaia Xingu | 45 | 56 | 647.808 | 74 | 28 | 731.321 |
| Soul | 1.312 | 636 | 3.978.292 | 1.784 | 461 | 4.170.510 |
| North Araguaia Karajá | 9 | 18 | 184.447 | 28 | 9 | 205.102 |
| Teles Pires | 680 | 331 | 2.787.891 | 598 | 332 | 3.029.380 |
| Middle North | 394 | 241 | 1.805.230 | 504 | 176 | 1.915.727 |
| **All** | **7.754** | **3.915** | **26.865.825** | **9.904** | **2.994** | **28.036.688** |
