# Supplementary material for: Trend in cancer incidence in Mato Grosso and its health regions, Brazil, 2001–2018
Source: Arch Public Health. 2025 Apr 1;83:87. doi: 10.1186/s13690-025-01503-9 (PMC11960033; doi:10.1186/s13690-025-01503-9)
Supplement: Supplementary file 4 — Supplementary Material 4 [file 13690_2025_1503_MOESM4_ESM.docx]

**SI-4.** Trends in cancer incidence, age-standardized, by world standard population, among women and men, by Health Region of the state of Mato Grosso, Brazil, 2001 – 2018

**
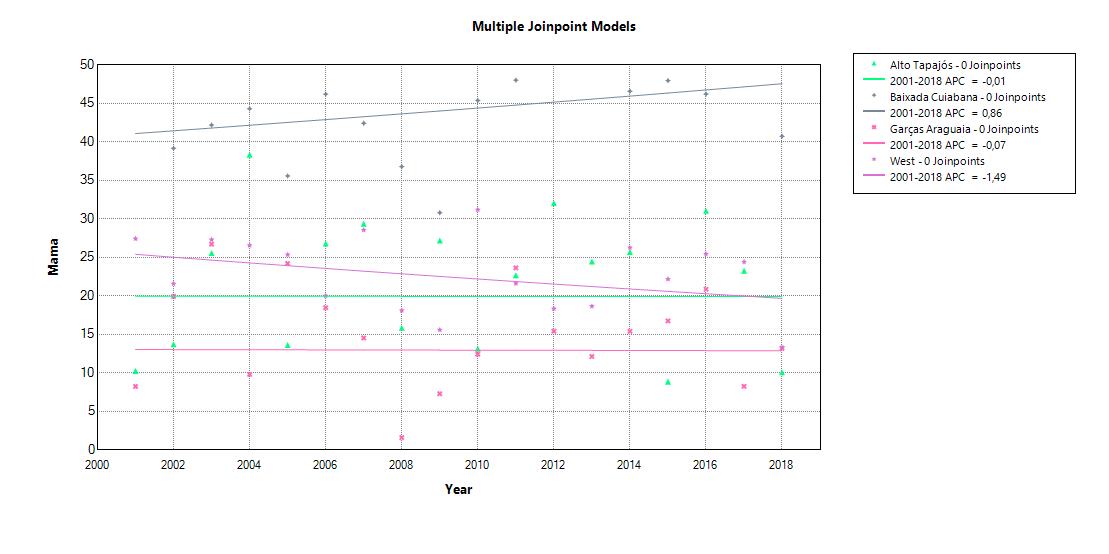

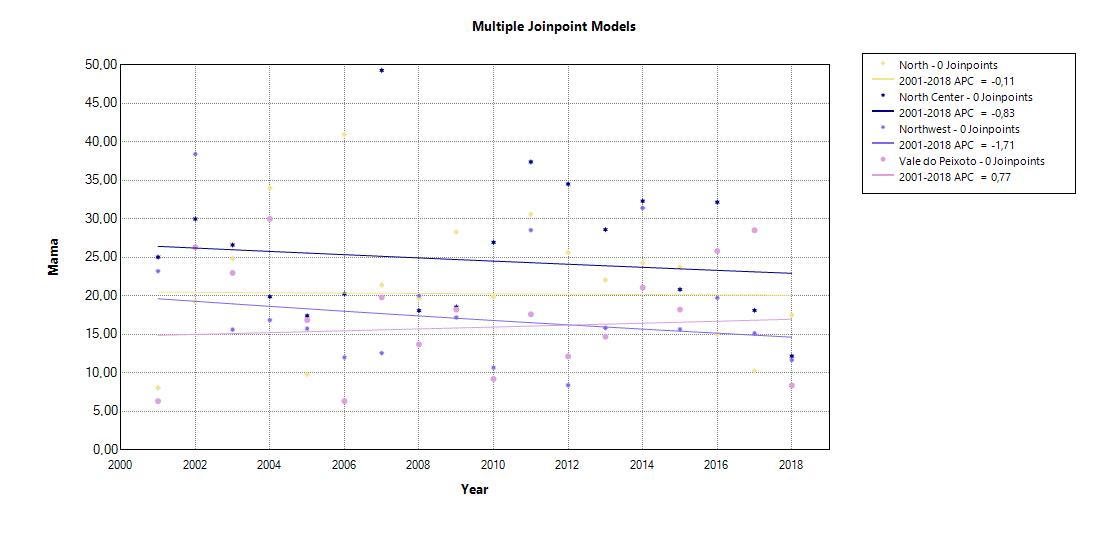

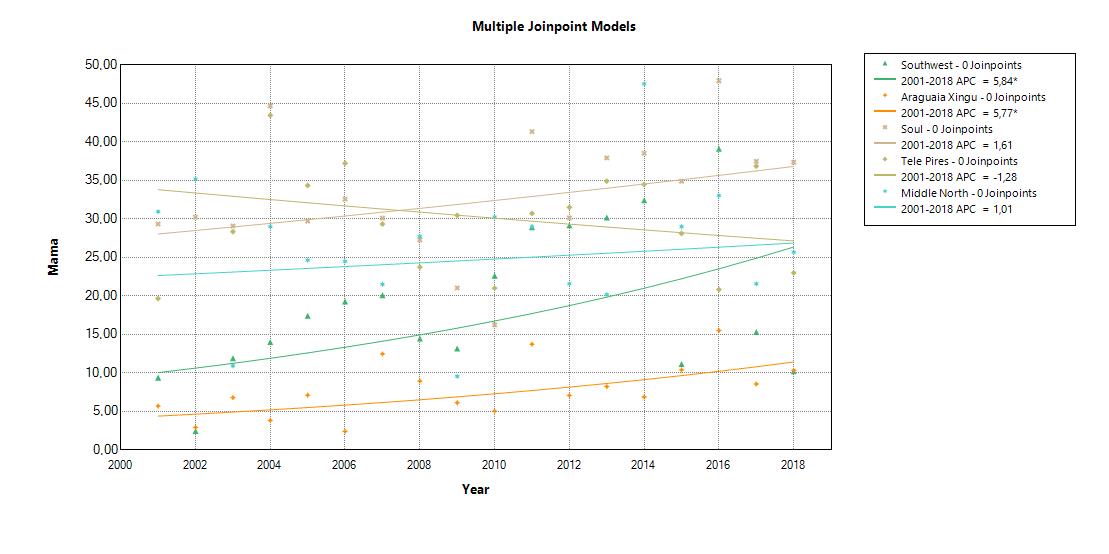
**

*APC or AAPC statistically significant (p<0.05).

**SI-4.1.** Trends in breast cancer incidence, age-standardized, by world standard population, among women, by Health Region of the state of Mato Grosso, Brazil, 2001 – 2018

**
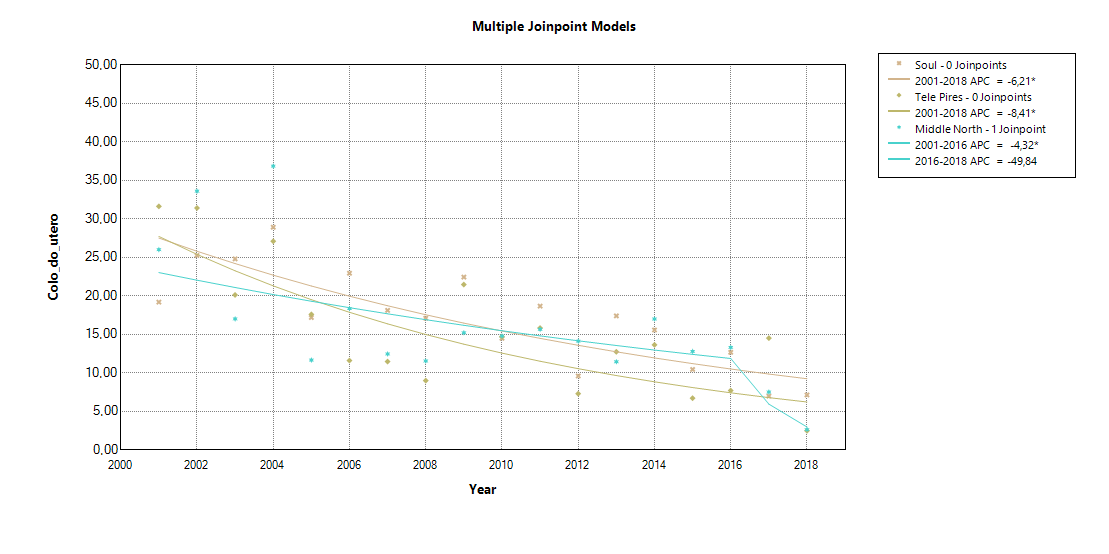

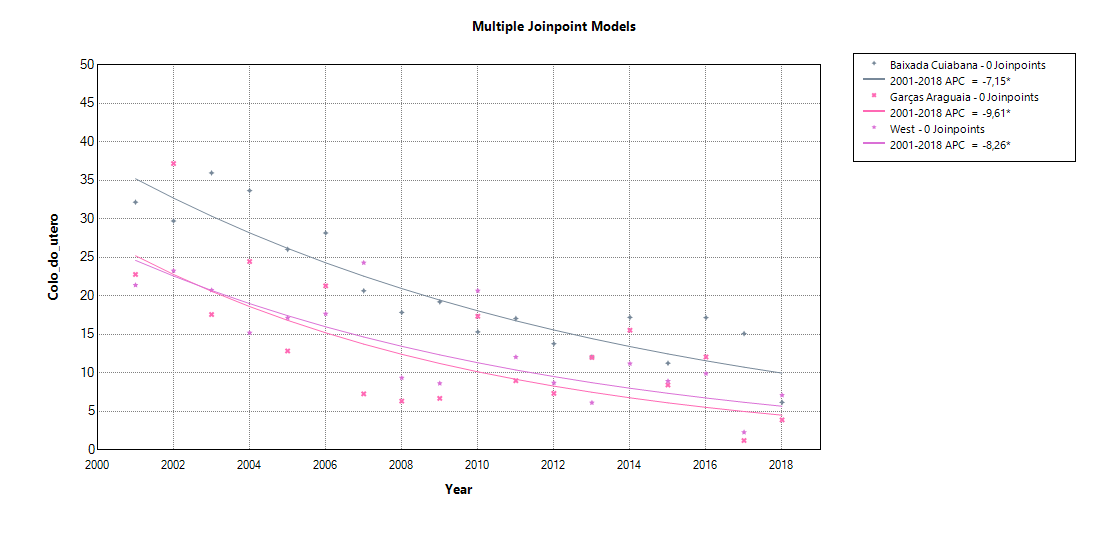

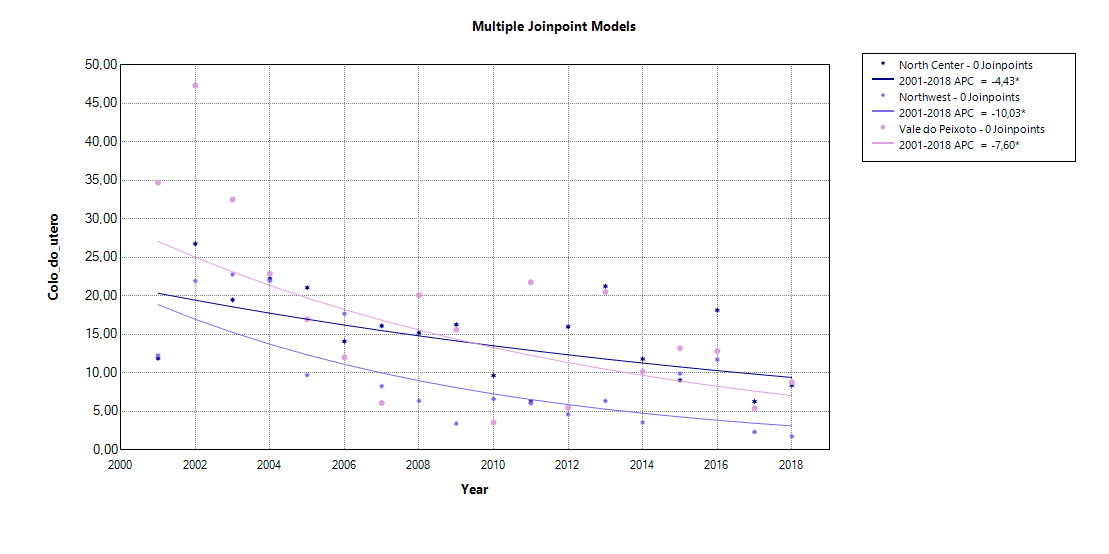
**

*APC or AAPC statistically significant (p<0.05).

**SI-4.2.** Trends in cervical cancer incidence, age-standardized, by world standard population, among women, by Health Region of the state of Mato Grosso, Brazil, 2001 – 2018

**
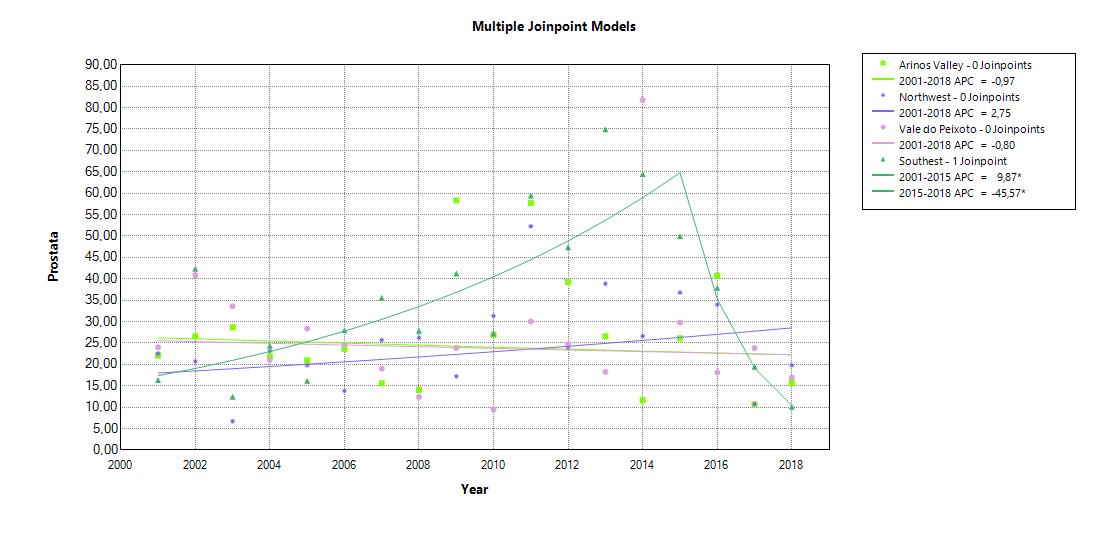
**

**
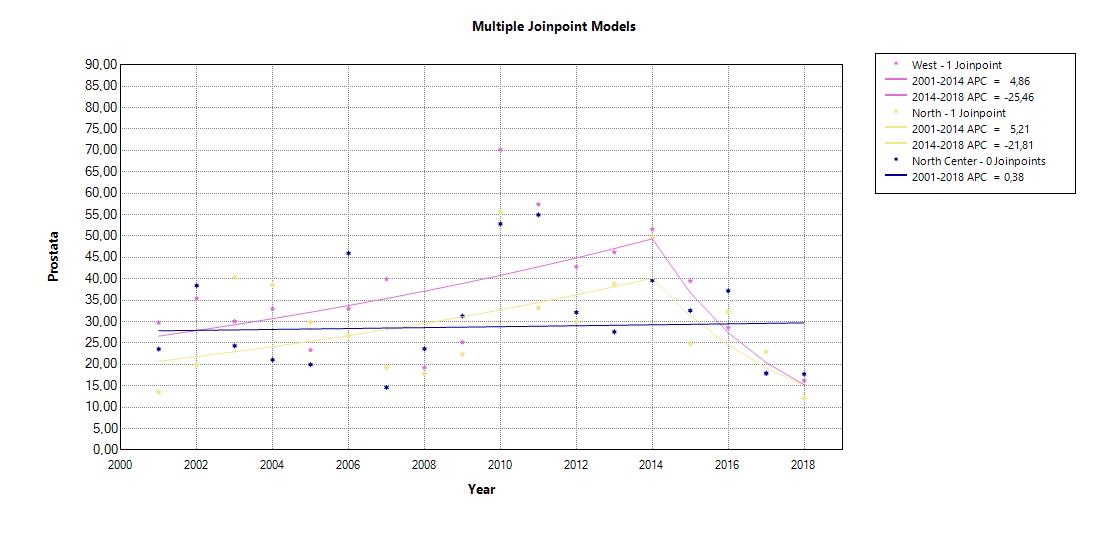

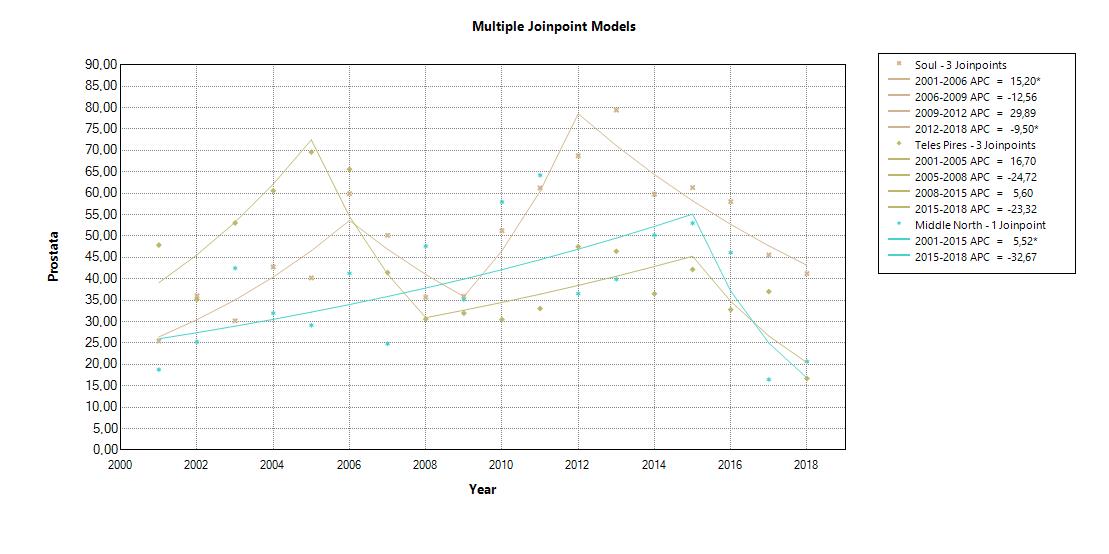

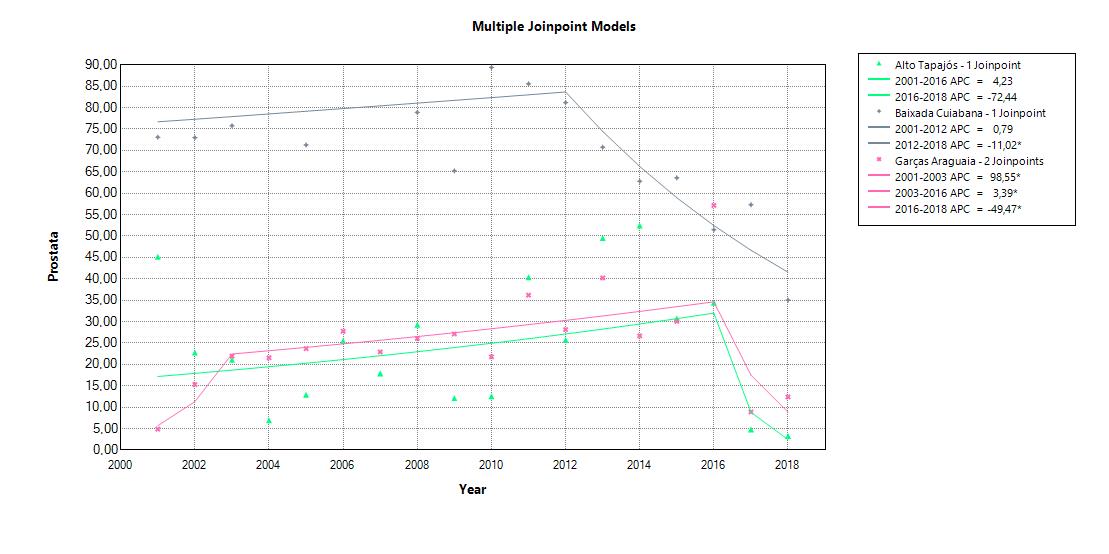
**

*APC or AAPC statistically significant (p<0.05).

**SI-4.3.** Trends in Prostate cancer incidence trends, age-standardized, by world standard population, among men, by Health Region of the state of Mato Grosso, Brazil, 2001 - 2018.


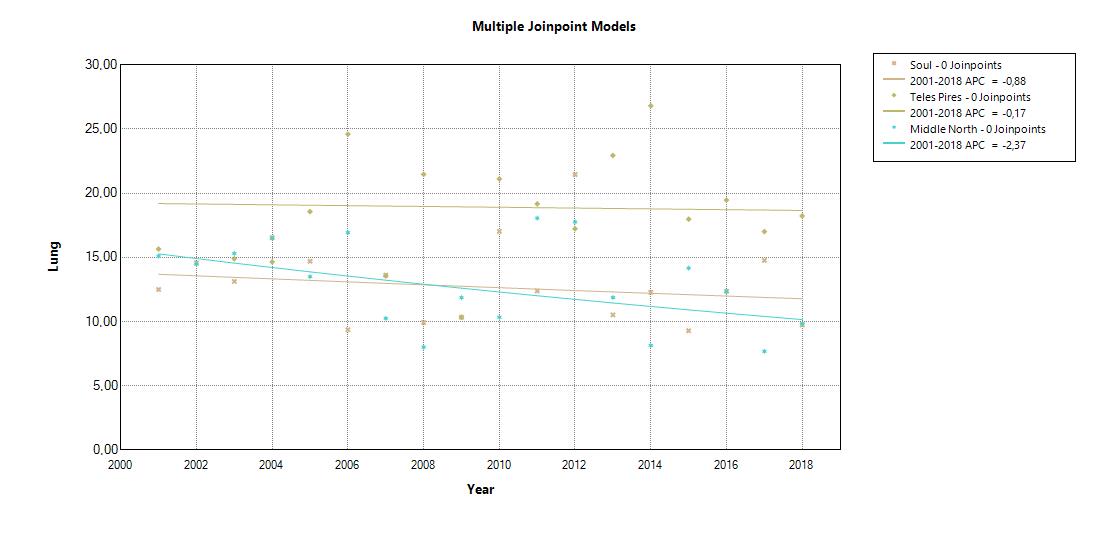

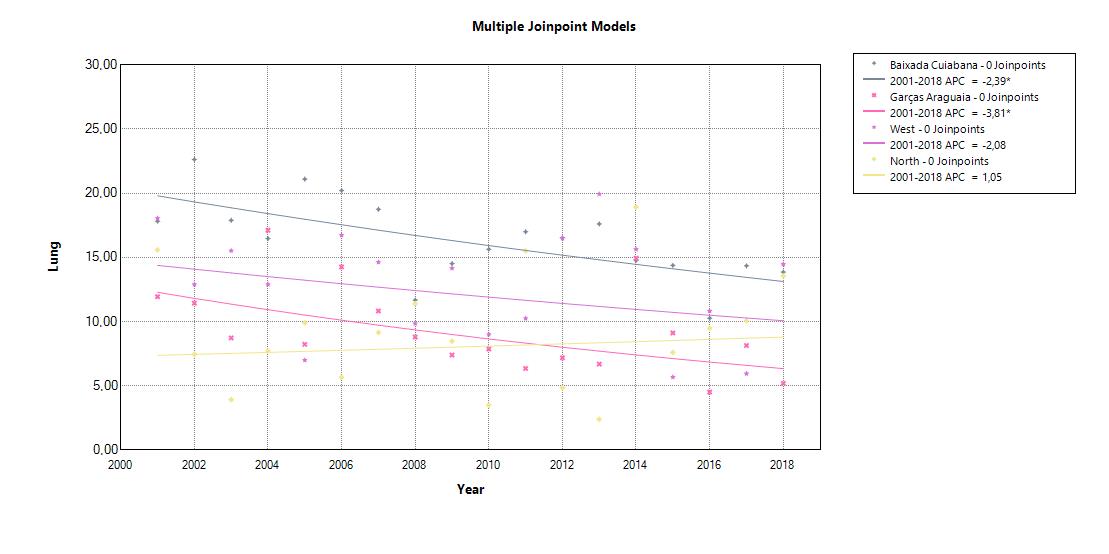

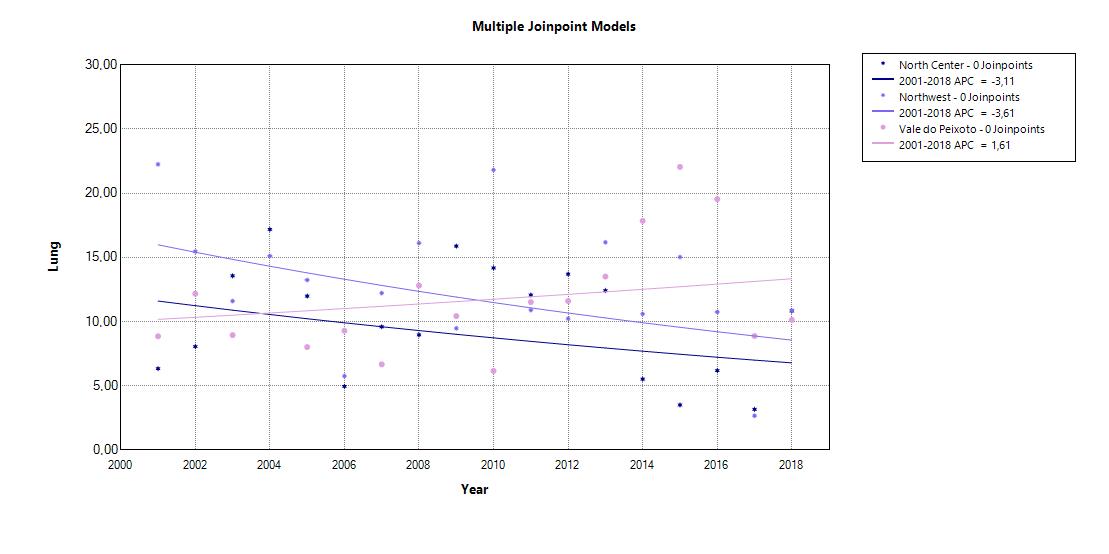


*APC or AAPC statistically significant (p<0.05)

**SI-4.4.** Trends in Lung cancer incidence trends, age-standardized, by world standard population, among men, by Health Region of the state of Mato Grosso, Brazil, 2001 - 2018
