# Supplementary material for: Trend in cancer incidence in Mato Grosso and its health regions, Brazil, 2001–2018
Source: Arch Public Health. 2025 Apr 1;83:87. doi: 10.1186/s13690-025-01503-9 (PMC11960033; doi:10.1186/s13690-025-01503-9)
Supplement: Supplementary file 5 — Supplementary Material 5 [file 13690_2025_1503_MOESM5_ESM.docx]

**SI-5.** Primary and secondary prevention measures according to cancer type.

| **CANCER** | **LEVEL OF PREVENTION** | |
| --- | --- | --- |
|  | **PRIMARY** | **SECONDARY** |
| PROSTATE | Promotion of healthy lifestyles that contribute to coping with overweight and obesity  - Healthy diet  - Regular physical activity (aerobic and resistance exercises) | Implementation of early detection practices:  - Early diagnosis - approach to people with early signs and/or symptoms of the disease  - Regular screening - prostate-specific antigen dosage and clinical examinations |
|  | Health education – implementation of awareness campaigns on risk factors | Immediate treatment |
|  | Professionals' performance in raising awareness of the target population about the importance of screening | Implementation of public policies that ensure quick and easy access to health services |
|  | Professionals adequately trained to implement primary and secondary prevention measures |  |
| LUNG | Reduction of Smoking and Passive Exposure through:  - Tobacco control policy and policies that promote smoke-free environments – legislative and social changes  - Awareness campaigns about the risks of smoking and the importance of regular medical appointments | Implementation of early detection practices through:  - Identification of signs and symptoms as early as possible (hemoptysis, cough, persistent hoarseness, chest pain, dyspnea, asthenia and weight loss with no apparent cause);  - Screening through imaging tests, such as CT scans in at-risk populations (smokers and former smokers) |
|  | Reduction of Exposure to air pollutants and carcinogens (asbestos, silica, uranium, chromium, alkylating agents, radon among others), drinking water containing arsenic |  |
|  | Promotion of healthy lifestyles  - healthy diet  - regular activity (aerobic and resistance exercises) and balanced diet |  |
|  | Reduce the occurrence of recurrent lung infections |  |
| BREAST | Implement actions that contribute to the control of modifiable risk factors and the promotion of protective factors | Implementation of early detection practices  - Regular clinical examinations, mammograms and the practice of breast self-examination. |
|  | Promotion of a healthy lifestyle (behavioral factors):  - Maintain adequate body weight  - Reduce alcohol consumption  – Regular physical activity  - Adopt a healthy/balanced diet  - Increase breastfeeding time  - Do not smoke and avoid passive smoking | Implementation of public policies that ensure quick and easy access to health services |
|  | Implementation of measures to address environmental risk factors:  - Reduce exposure to ionizing radiation, pesticides, benzene, dioxins, volatile organic compounds, magnetic and low-frequency electromagnetic fields,  - Avoid night work; |  |
|  | Implementation of health education practices, aiming to**:**  **-** Awareness of modifiable risk factors  - Access to information and awareness about the importance of screening tests, and  - Performance of health professionals - in educating patients about the importance of self-care and in carrying out screening exams |  |
|  | Professionals adequately trained to implement primary and secondary prevention measures |  |
| CERVICAL | Implement vaccination against the HPV virus (Human Papillomavirus) | Implementation of early detection practices:  - Regular screening exams, (Pap smear)  - Existence of organized and accessible tracking programs |
|  | Implementation of health education practices aiming to:  - Raise awareness of the importance of vaccination and prevention practices  - Overcome cultural and social barriers that hinder adherence to preventive exams | Treatment of precursor lesions |
|  | Promotion of safe sex practices | Implementation of public policies that ensure quick and easy access to health services |
|  | Professionals adequately trained to implement primary and secondary prevention measures |  |

Source: Synthesis prepared by the authors based on the references used in this article.
